# Anesthetic Challenges in an Infant With Tetralogy of Fallot Posted for Non-cardiac Surgery: A Case Report and Literature Review

**DOI:** 10.7759/cureus.47327

**Published:** 2023-10-19

**Authors:** Neeta Verma, Sheetal Madavi, Jui A Jadhav, Sambit Dash, Aruna Chandak

**Affiliations:** 1 Department of Anesthesiology, Jawaharlal Nehru Medical College, Acharya Vinoba Bhave Rural Hospital, Datta Meghe Institute of Higher Education & Research (Deemed to Be University), Wardha, IND

**Keywords:** regional anesthetic, pulmonary hypertension, non-cardiac surgery, left to right shunt, fontan physiology, congenital heart disease

## Abstract

The goal of this article is to provide an up-to-date and comprehensive review of the current perioperative anesthetic management of pediatric patients with congenital heart disease (CHD) undergoing non-cardiac surgery. This report discusses a case of a nine-month-old female with Tetralogy of Fallot who was scheduled for non-cardiac surgery for anorectal malformation stage 1 and stage 2 repair. This case study discusses how to adjust perioperative anesthesia care in cases of left-to-right shunt, right-to-left shunt, and complex cardiac disease. In addition, the author discusses special considerations such as pulmonary hypertension, newborns with CHD undergoing extracardiac surgery, and the importance of regional anesthesia in children with CHD undergoing non-cardiac surgery.

## Introduction

Around one in every 125 live births is affected by congenital heart disease (CHD) [1]. About 10% of cases of CHD are attributed to Tetralogy of Fallot (TOF) [2]. Because of its existence, the perioperative risk and mortality rate are increased. The preoperative planning for surgery, the administration of anesthesia during surgery, and the treatment of frequent postoperative difficulties in the critical care unit are all examples of perioperative considerations that must be made for these patients [3].

## Case presentation

**Figure 1:**
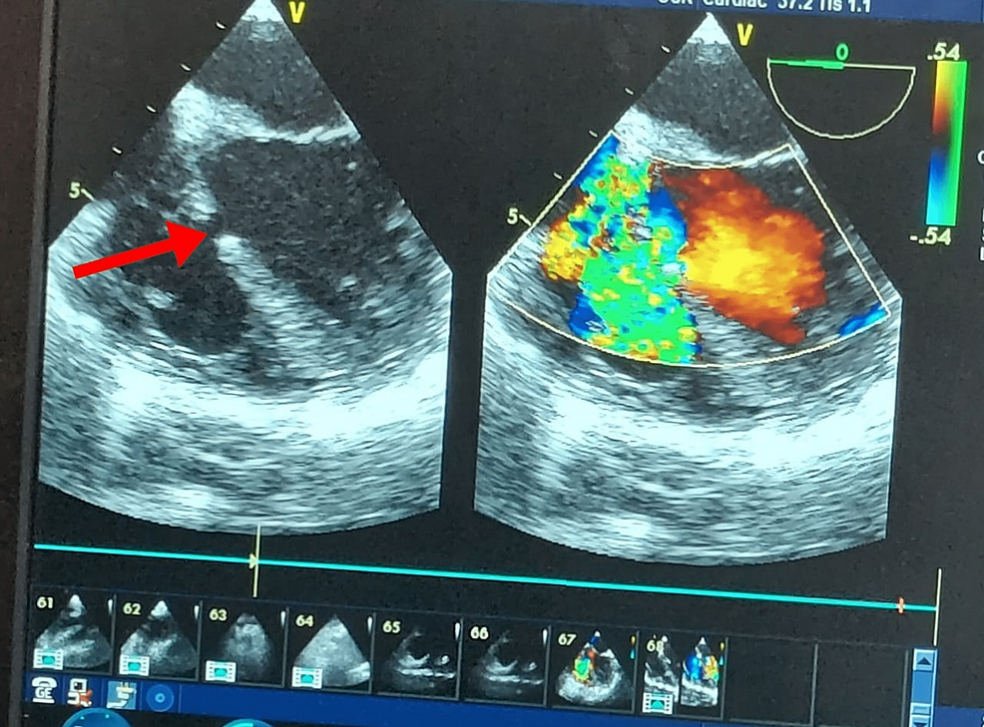
2D echocardiography monitor image showing large ventricular septal defect.

A nine-month female term baby, weighing 2.7 kg, delivered by normal vaginal delivery, with a two-day history of neonatal intensive care unit (ICU) stay due to dyspnea, presented to casualty with complaint of stool in urine. The patient also gives a history of intermittent symptoms of dyspnea and shortness of breath. After performing basic routine investigations like complete blood count, kidney function test, liver function test, urine routine microscopy and culture, and chest X-ray, the patient was referred to the pediatric surgery department for further course of action. On detailed general examination and history taking, the patient was found to be a known case of TOF with 2D echocardiography showing large subaortic ventricular septal defect (Figure 1), severe hypoplastic pulmonary artery, moderate patent ductus arteriosus, with left-to-right shunt and multiple collaterals. She was referred to a pediatric cardiologist and cardiothoracic and vascular surgeon (CTVS) for their opinion. CTVS surgeon opined that cardiac surgery should be performed first as it is a priority. However, due to the complexity of the surgery and lack of consent of parents, anorectal malformation (ARM) stage 1 and stage 2 repair in a single sitting was planned. Cardiac anesthesiologists were also notified about the case. Parents were clearly informed about the risks involved in surgery and anesthesia.

**Figure 2:**
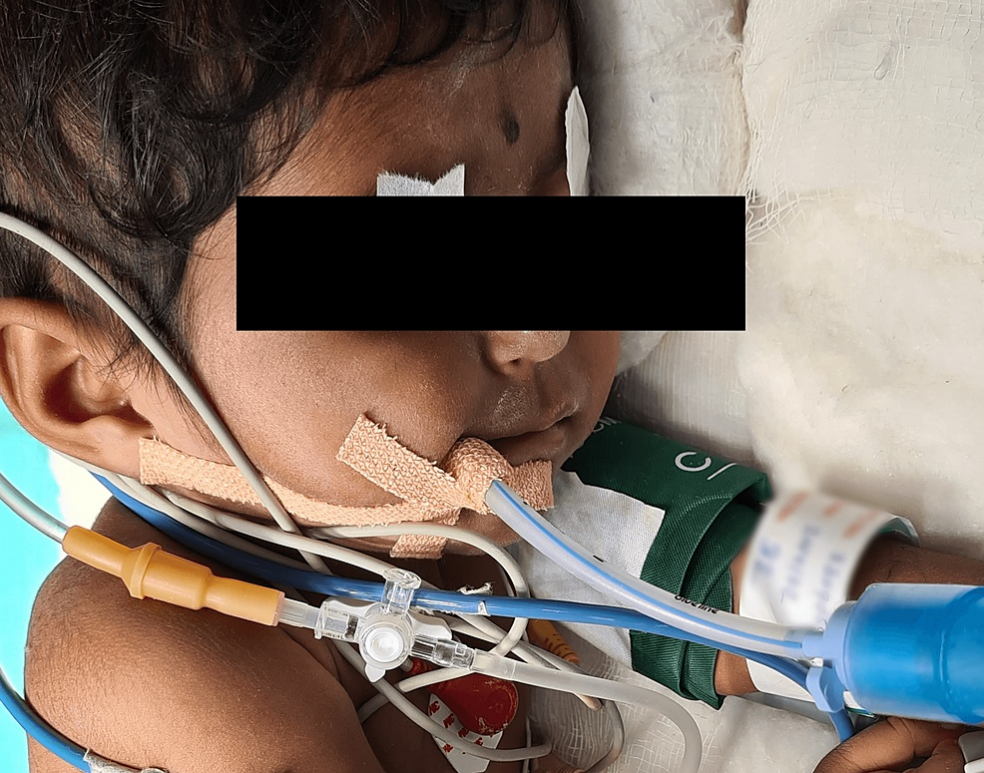
Image showing the patient after the airway was secured with a 4 mm uncuffed endotracheal tube.

**Figure 3:**
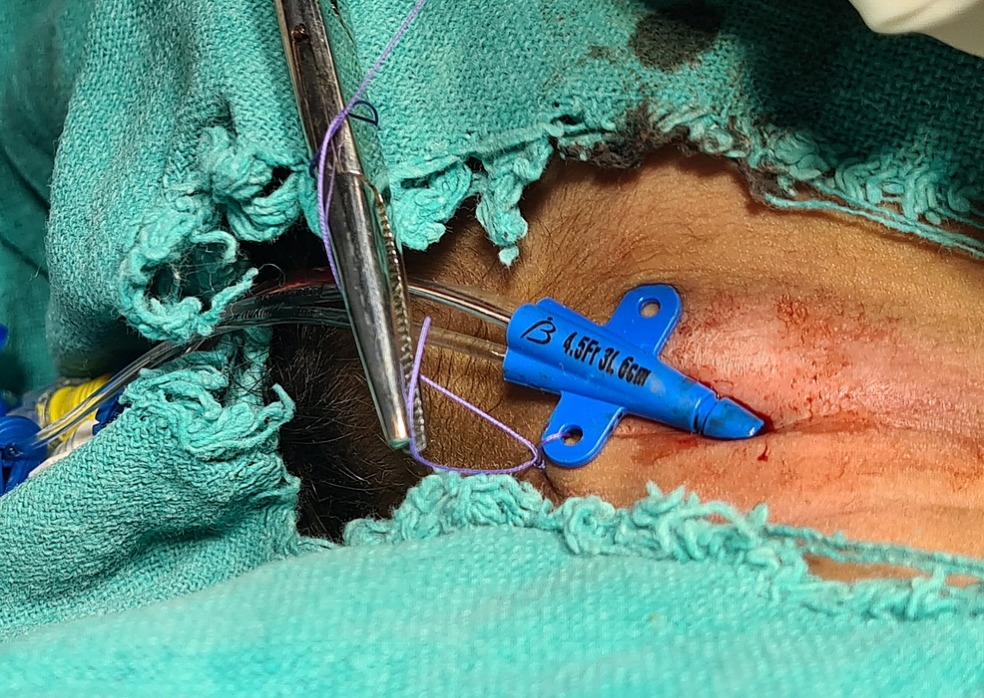
Image showing the 4.5 Fr triple lumen central venous catheter secured in the right internal jugular vein.

During a preanesthetic check-up (PAC), the baby was found to be active. There was no active complaint of dyspnea, and the room air peripheral oxygen saturation (SpO_2_) was 84%. She had no other associated congenital anomalies. Birth history was by normal vaginal delivery, term baby. There was no history of neonatal ICU stay. The baby was immunized up to age and had no developmental milestone delay. On clinical examination, mouth opening was adequate, and the chest was bilaterally clear with no added sounds on auscultation. Ejection systolic murmur was elicited. The hemoglobin of the patient was found to be low at 7.8 gm/dl, and the patient had visible pallor on examination. Fitness for surgery was given under high risk after detailed written and verbal consent from parents. On the day of surgery, after connecting American Society of Anesthesiologists (ASA) standard monitors, a 24G peripheral vascular catheter was secured in the right upper arm and general anesthesia was administered with Inj. glycopyrolate 0.004 mg/kg, Inj. midaz 0.05 mg/kg, Inj. fentanyl 2 mcg/kg, Inj. etomidate 0.3 mg/kg, Inj. atracurium 0.5 mg/kg, and a 4 mm uncuffed endotracheal tube was secured (Figure 2). After securing the tube, a 4.5 Fr triple lumen central venous catheter was secured, which is ultrasonography-guided, in the right internal jugular vein and was secured after confirmation of venous blood via venous blood gas analysis (Figure 3). The arterial line was secured in the right radial artery with a 24G peripheral vascular catheter for invasive blood pressure monitoring and for drawing arterial blood gas (ABG) at regular intervals. Anesthesia was maintained on intermittent doses of injection atracurium 0.5 mg every 15-20 minutes and sevoflurane 2%.

**Figure 4:**
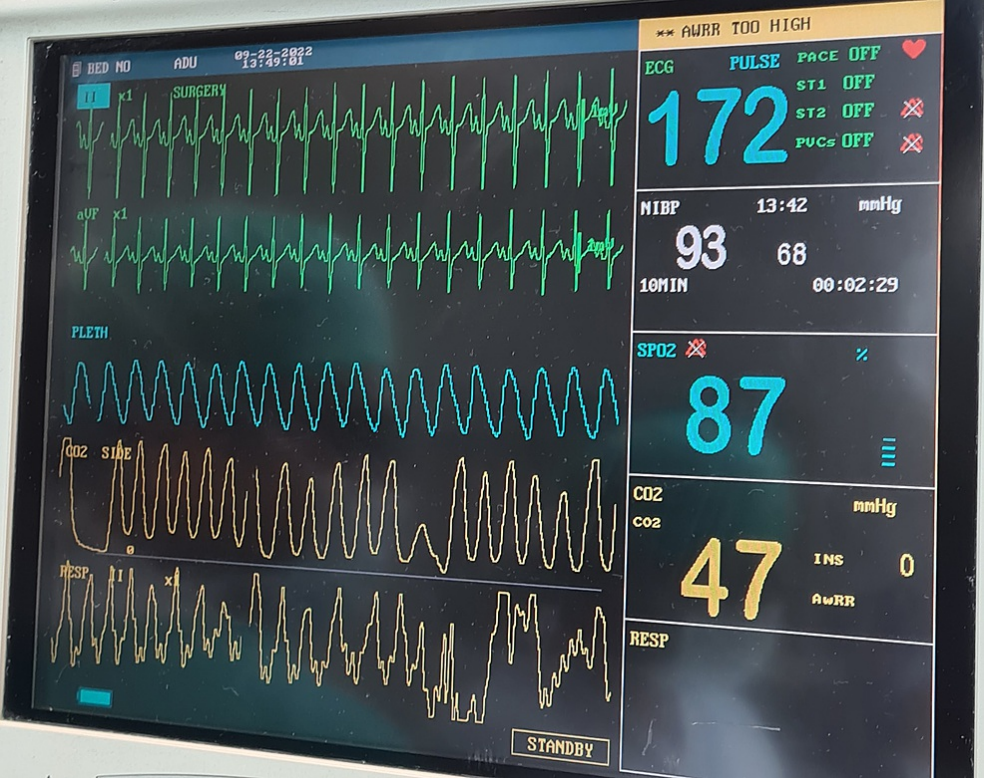
Image showing the intraoperative vitals of the patient.

**Figure 5:**
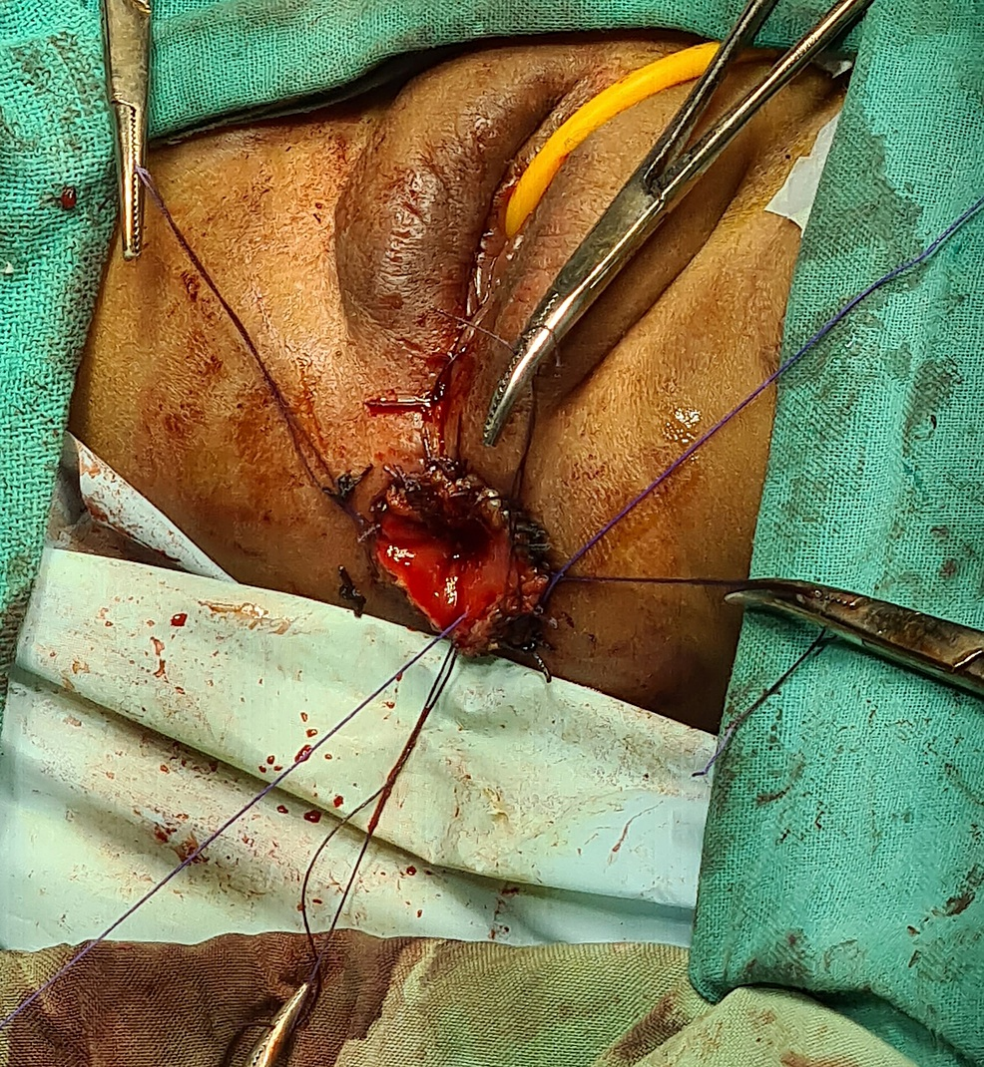
Image showing the intraoperative image of anorectal malformation repair.

Oxygenation was maintained at a fraction of inspired oxygen (FiO_2_) of 60%. Calculated fluid was given according to the Holliday-Segar scale and strict input-out monitoring was done. Fluid overload was avoided. ABG was sent every two hours and corrections were made accordingly. About 40 ml of packed red blood cells were administered slowly over two hours. Injection esmolol at 0.5 mg/kg was used to counter any intraoperative arrhythmia. Hemodynamics were maintained throughout (Figure 4). The rise in systemic vascular resistance was avoided. Hypoxia and hypercarbia were avoided and the FiO_2_ was maintained on the higher side. Anesthesia was maintained with a minimum alveolar concentration of 0.8-0.9 of sevoflurane. ARM stage 1 and stage 2 repair was done in the same setting to avoid putting the patient twice under surgical stress (Figure 5).

At the end of the three-hour surgery, the patient was reversed with neostigmine and atropine combination (Myo Pyrolate) at a dose of 0.05 mg/kg. Post reversal, after assessing the cough reflex and alertness of the child, the patient was extubated and 100% oxygen was administered to the patient for five minutes. Breathing and room air oxygen saturation were checked for at least 5 to 10 minutes before shifting the patient to the pediatric ICU. The chest was auscultated and found to be clear without any added sounds. The patient was vigilantly monitored for any sign of distress at the ICU.

## Discussion

In patients who have cyanotic heart disease, the aims of anesthesia are to maintain or raise systemic vascular resistance (SVR), reduce pulmonary vascular resistance (PVR), and avoid hypercyanotic episodes while the patient is undergoing surgery [4]. Because of the potential for protracted induction and the ease with which the patient might have cyanotic episodes owing to an imbalance of SVR to PVR, general anesthesia was not our first option for the patient. They may also have arrhythmias, hypovolemia, hypothermia, and other conditions that, when left untreated, may lead to cyanosis. Throughout the intraoperative and postoperative periods, it was important to prevent metabolic disturbances such as hypovolemia, hypoxemia, hypo/hypercapnia, hypo/hyperthermia, and hypo/hyperglycemia, among others. When treating patients who have TOF, it is important to avoid tachycardia, dehydration, and sympathetic stimulation because these factors can cause spasms of hypertrophied pulmonary infundibulum, which can result in the development of hypercyanotic spells or tet spells. These spells are characterized by rapidly falling SpO_2_ levels, hypotension, and ischemic ECG changes.

Patients with CHD who are going to have non-cardiac surgery are still considered to be in the high-risk group after the procedure, even if the results are favorable; thus, these patients should be monitored in a high-dependency unit or ICU [5]. When it comes to intraoperative or postoperative care, pain management is a very important issue. In addition to having analgesic effects and keeping these patients in a safe environment, it reduces the sympathetic outflow from the central nervous system in a dose-dependent way [6].

## Conclusions

Patients with congenital cardiac issues like TOF present with multiple risks and pose great challenges for any anesthesiologist in the smooth induction and maintenance of anesthesia. Special considerations need to be taken like securing a central venous catheter, securing an invasive arterial line catheter for continuous blood pressure monitoring, and repeated ABG samples. The extubation should not be hurried and post-operative monitoring and care become more crucial. Emergency cardiac drugs should be kept ready and any untoward cardiac event or arrhythmias should be taken care of without haste.
